# Comparative transcriptome and histomorphology analysis of testis tissues from mulard and Pekin ducks

**DOI:** 10.5194/aab-63-303-2020

**Published:** 2020-09-08

**Authors:** Li Li, Linli Zhang, Zhenghong Zhang, Nemat O. Keyhani, Qingwu Xin, Zhongwei Miao, Zhiming Zhu, Zhengchao Wang, Junzhi Qiu, Nenzhu Zheng

**Affiliations:** 1 College of Life Sciences, Fujian Agriculture and Forestry University, Fuzhou 350002, China; 2 Institute of Animal Husbandry and Veterinary Medicine, Fujian Academy of Agricultural Sciences, Fuzhou 350013, China; 3 College of Life Sciences, Fujian Normal University, Fuzhou 350007, China; 4 Department of Microbiology and Cell Science, Institute of Food and Agricultural Sciences, University of Florida, Gainesville, FL 32611, USA

## Abstract

Testicular transcriptomes were analyzed to characterize the differentially expressed genes between mulard and Pekin ducks, which will help establish gene expression datasets to assist in further determination of the mechanisms of genetic sterility in mulard ducks. Paraffin sections were made to compare the developmental differences in testis tissue between mulard and Pekin ducks. Comparative transcriptome sequencing of testis tissues was performed, and the expression of candidate genes was verified by quantitative reverse transcription-polymerase chain reaction (qRT-PCR). In mulard ducks, spermatogonia and spermatocytes were arranged in a disordered manner, and no mature sperm were observed in the testis tissue. However, different stages of development of sperm were observed in seminiferous tubules in the testis tissue of Pekin ducks. A total of 43.84 Gb of clean reads were assembled into 193 535 UniGenes. Of these, 2131 transcripts exhibited differential expression (false discover rate <0.001 and fold change ≥2), including 997 upregulated and 1134 downregulated transcripts in mulard ducks as compared to those in Pekin duck testis tissues. Several upregulated genes were related to reproductive functions, including ryanodine receptor 2 (RYR2), calmodulin (CALM), argininosuccinate synthase and delta-1-pyrroline-5-carboxylate synthetase ALDH18A1 (P5CS). Downregulated transcripts included the testis-specific serine/threonine-protein kinase 3, aquaporin-7 (AQP7) and glycerol kinase GlpK (GK). The 10 related transcripts involved in the developmental biological process were identified by GO (Gene Ontology) annotation. The KEGG (Kyoto Encyclopedia of Genes and Genomes) pathways indicated that peroxisome proliferator-activated receptors (PPARs) and calcium signaling pathways were significantly (P<0.001) associated with normal testis physiology. The differential expression of select genes implicated in reproductive processes was verified by qRT-PCR, which was consistent with the expression trend of transcriptome sequencing (RNA-seq). Differentially expressed candidate genes RYR2, CALM, P5CS, AQP7 and GK were identified by transcriptional analysis in mulard and Pekin duck testes. These were important for the normal development of the male duck reproductive system. These data provide a framework for the further exploration of the molecular and genetic mechanisms of sterility in mulard ducks.

**Highlights.** The mulard duck is an intergeneric sterile hybrid offspring resulting from mating between Muscovy and Pekin ducks. The transcriptomes of testis tissue from mulard and Pekin ducks were systematically characterized, and differentially expressed genes were screened, in order to gain insights into potential gonad gene expression mechanisms contributing to genetic sterility in mulard ducks.

## 1 Introduction

The mulard duck is a famous local variety in China and is considered a highly prized delicacy. The duck is the intergeneric hybrid progeny of a male Muscovy duck (*Cairina moschata* L.) and female domestic duck (*Anas platyrhynchos* var. *domestica*). The mulard duck exhibits strong heterosis, including high resistance, a high feed reward and delicate/high meat quality compared with its parents. However, there were no significant differences between the appearance of males and females, no obvious sex differentiation and sexual behaviors, and no actual seed values able to be determined for mulard ducks, which are essentially nonreproductive and considered sterile.

However, a careful examination revealed the presence of testes in nominally male mulard ducks during the breeding process, which produced a small amount of semen, indicating that these ducks may not be completely sterile. Indeed, on occasion, individuals have been identified as displaying various aspects of normal male mating, intromission or female egg laying. These findings suggest that one reason for the nonreproductive nature of the mulard duck may be related to the regulation of gene expression and cell differentiation in their reproductive organs because distantly hybridized sterility is a very complex biological phenomenon whose genetic basis is likely regulated by a diverse range of genetic pathways. Our previous research indicated that inconsistent karyotypes from parents contributed to intergenerically hybridized sterility because of incorrect meiosis and poor gonadal development (Tan et al., 1998). At present, transcriptome sequencing (RNA-Seq) technology has been widely applied to the detection of differential gene expression and functional annotation in livestock and poultry (Chen et al., 2016; Cong et al., 2013; Liu et al., 2017; Zeng et al., 2015). This has included investigations of reproductive mechanisms of genes related to follicular development in ducks (Xu et al., 2013) and differential gene expression in the ovaries of Shan Ma ducks, which compared peak laying and late laying periods (Zhu et al., 2017), as well as the examination of differentially expressed miRNAs between laying and non-laying periods (Yu et al., 2013). However, to date, differential gene expression has not been used to examine the genetic mechanism that may contribute to the sterility traits and reproductive performance of mulard ducks.

Given the particularity of sterility in mulard ducks, the present study utilized RNA-Seq technology to compare the transcriptomes in the testes of mulard and Pekin ducks. The set of differentially expressed genes (DEGs) were screened and characterized via the Gene Ontology (GO) and the Kyoto Encyclopedia of Genes and Genomes (KEGG) databases for functional annotation and analysis to identify genes and genetic pathways related to the sex dysplasia phenotype of mulard ducks. These data will provide the basis for further investigation of the abnormal differentiation mechanisms in the reproductive system of mulard ducks and provide a framework for hypothesis development that could potentially lead to insights into treating or alleviating mulard duck infertility.

## 2 Materials and methods

### 2.1 Animals

The experimental animals included male mulard and Pekin ducks, which were housed for 3–6 months with standardized feeding regulations in the Laboratory Animal Center, Fujian Academy of Agricultural Sciences (Fujian, China). The experimental protocol was approved by the Institutional Animal Care and Use Committee and the Ethics Committee on Animal Experimentation, Fujian Academy of Agricultural Sciences. The ethics committee approval number is FAAS-IAHV-AEC-2017-0510.

Experimental ducks were used for collecting semen at sexual maturity (180 d old). Animals were fasted for 12 h at the age of 210 d and then immediately sacrificed for sample (dissection of the testes) collection. The animals used included mulard ducks that did not show mounting/sexual behavior and Pekin ducks that exhibited normal mounting behaviors. Dissected testes were immediately placed in liquid nitrogen and stored at -80 ${}^{\circ }$C until further processing.

### 2.2 Gonadal sections of mulard ducks and Pekin ducks

Two mulard and Pekin ducks were examined, and the development of testes was observed after dissection. The testis tissues were fixed in 10 % formaldehyde solution, embedded in paraffin and stained with hematoxylin and eosin to create tissue sections.

### 2.3 RNA extraction and library preparation for transcriptome sequencing

Two individuals from each variety were used for transcriptome sequencing and passed the biological repeat test, which indicated that the samples used in this study exhibited good biological repeatability and reasonable sample grouping and met the requirements of transcriptome sequencing; consequently, no additional materials were added. Total RNA was isolated from the testes using the RNeasy Lipid Tissue Mini Kit (QIAGEN, Germany) following the manufacturer's protocol. Sequencing libraries were generated using the NEBNext^®^ Ultra™ RNA Library Prep Kit for Illumina^®^ (NEB, USA) analysis following the manufacturer's recommendations, and barcodes were added to attribute sequences to each sample. To select cDNA fragments of ∼ 150–200 bp in length, the libraries were purified using the AMPure XP system (Beckman Coulter, Beverly, USA), followed by polymerase chain reaction (PCR) amplification. Library quality was assessed using the Agilent Bioanalyzer 2100 system.

### 2.4 Clustering and sequencing

Clustering of the barcoded samples was performed on a cBot Cluster Generation System using the TruSeq PE Cluster Kit v3-cBot-HS (Illumina) in accordance with the manufacturer's instructions. After cluster generation, the library preparations were sequenced on an Illumina HiSeq 2000 platform, and paired-end reads were generated. Transcriptome assembly was accomplished based on the left fq and right fq using Trinity to determine transcription and gene expression levels. Differential gene expression analyses of the two conditions/groups (mulard versus Pekin) were performed using the DESeq R package (1.10.1). Transcript expression levels with an adjusted P value of <0.05 determined by DESeq were designated as differentially expressed.

### 2.5 GO and KEGG pathway enrichment analysis enrichment analysis

GO enrichment analyses of the DEGs were implemented using the top GO R packages based on the Kolmogorov–Smirnov test. DEGs were classified and analyzed by the Clusters of Orthologous Groups and KEGG databases, and P<0.05 was used as the significance enrichment standard.

### 2.6 Quantitative reverse transcription-polymerase chain reaction

Quantitative reverse transcription-polymerase chain reaction (qRT-PCR) was performed to validate four DEGs in the cDNA pools composed of two individuals from each group. The qRT-PCR primers were designed by Primer Premier 6 and Beacon Designer 7.8 and then synthesized by Bioengineering Co., Ltd. (Shanghai). The primer sequences are listed in Table 1. The qRT-PCR was run at 95 ${}^{\circ }$C for 1 min, followed by 40 cycles at 95 ${}^{\circ }$C for 15 s and 63 ${}^{\circ }$C for 25 s (fluorescence collection). The qRT-PCR was performed in a 20 L reaction mixture containing 1 L cDNA template, 10 L PowerUp SYBR^®^ Green Master Mix (Applied Biosystems A25779), 8 µL sterile distilled water and 0.5 µL of each primer. The amount of target gene transcript relative to the internal control gene, β-actin, was calculated in accordance with the ΔΔCt method (Livak et al., 2001). Relative mRNA levels were reported as 2${}^{-\Delta \Delta \mathrm{Ct}}$ values. The results of three independent experiments were used for statistical analysis; P<0.05 was considered statistically significant.

**Table 1:** Primer information for qRT-PCR.

Gene	Full name	Primer sequences (5${}^{\prime }$ to 3${}^{\prime }$)	Product	Annealing
			size (bp)	(${}^{\circ }$)
ZP4	Zona pellucida sperm-binding protein 4	F: GGCTGTGGGCTCTGGGTTT	93	60
		R: GTTGCCATCCCATTCAAAGACAT		
CAMK4	Calcium/calmodulin-dependent protein kinase	F: GCAGAAAGGGACCCAGAAACCT	109	60
	type IV	R: GTTGGGATGTGAAAGGCGAAGA		
CPCX1	Centrosomal protein C10orf90 homolog	F: GCAAGAAAGGCTGAAGAAGCTG	86	60
	isoform X1	R: GCGCTCCTTGGTTGCTTACAG		
CALM	Calmodulin-like	F: CCGAGGAGCAGATTGCAGAGT	150	60
		R: CCTCGTTGATCATGTCCTGCA		
DS3	Delta-1-pyrroline-5-carboxylate synthase	F: CTGCATATAGCAATCAAATCCTTCA	85	60
	isoform X3	R: CTTACAAGTTGCACTGCATCTTTGA		
β-actin	Beta-actin	F: GATGTGGATCAGCAAGCAGGAGT	95	60
		R: GGGTGTGGGTGTTGGTAACAGT		

Note: F is forward primer; R is reverse primer.

## 3 Results

### 3.1 Testis tissue slices of mulard and Pekin ducks

**Figure 1:**
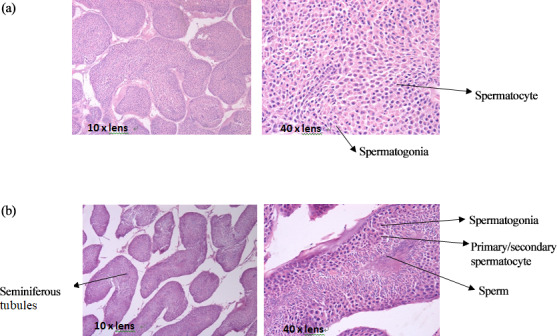
The paraffin slices of testis tissues from the mulard duck **(a)** and Pekin duck **(b)**.

The comparison of paraffin slices of testis tissues from mulard and Pekin ducks (Fig. 1) showed that the seminiferous tubule of the mulard duck was not obvious and had thick connective tissue. Spermatogonia and spermatocytes could be seen; however, the cells disordered, and no mature sperm were observed. The complete process of spermatogenesis was shown in the testis tissue sections of Pekin ducks. Different stages of the development of sperm were observed in the seminiferous tubules; cells close to the wall of the seminiferous tubules were oblate primitive cells, the large, round cells were primary/secondary spermatocytes, and, after meiosis, the mature haploid sperm cells were located at the center of the lumen. These reveal the differences in gonadal development of mulard and Pekin ducks on an apparent level.

### 3.2 Transcriptome sequencing results of testis tissues

Purified mRNA libraries derived from dissected testes isolated from mulard and Pekin ducks were constructed and sequenced as detailed in Sect. 2, yielding a total of 31.05 Gb of data. After data cleanup, ∼6.34 Gb of data for each condition (∼12.68 Gb total) was obtained, and the percentage of Q30 (clean data mass of not less than 30 bases) was above 91.36 % with a GC content (the percentage of G and C bases in the total bases in clean data) of ∼52.24 % (Table 2). Additionally, the ratio of mapped reads to transcripts or the UniGene was ∼68.73 %, indicating that the sequencing quality was reliable.

**Table 2:** Summary data of the sequencing assembly.

Sample name	Clean reads	Base number	% ≥ Q30	GC content	Mapped reads	Mapped ratio
T03	21 461 374	6 349 389 158	91.55 %	53.78 %	14 750 395	68.73 %
T04	29 719 432	8 829 987 620	91.40 %	52.24 %	20 682 985	69.59 %
T05	21 859 316	6 489 813 952	91.48 %	51.88 %	14 983 171	68.54 %
T06	21 217 125	6 293 982 810	91.36 %	52.43 %	14 681 967	69.20 %

Notes: T03 and T04 represent two biological replicates of Pekin ducks; T05 and T06 represent two biological replicates of mulard ducks. The same below.

### 3.3 Screening of DEGs and cluster analysis

**Figure 2:**
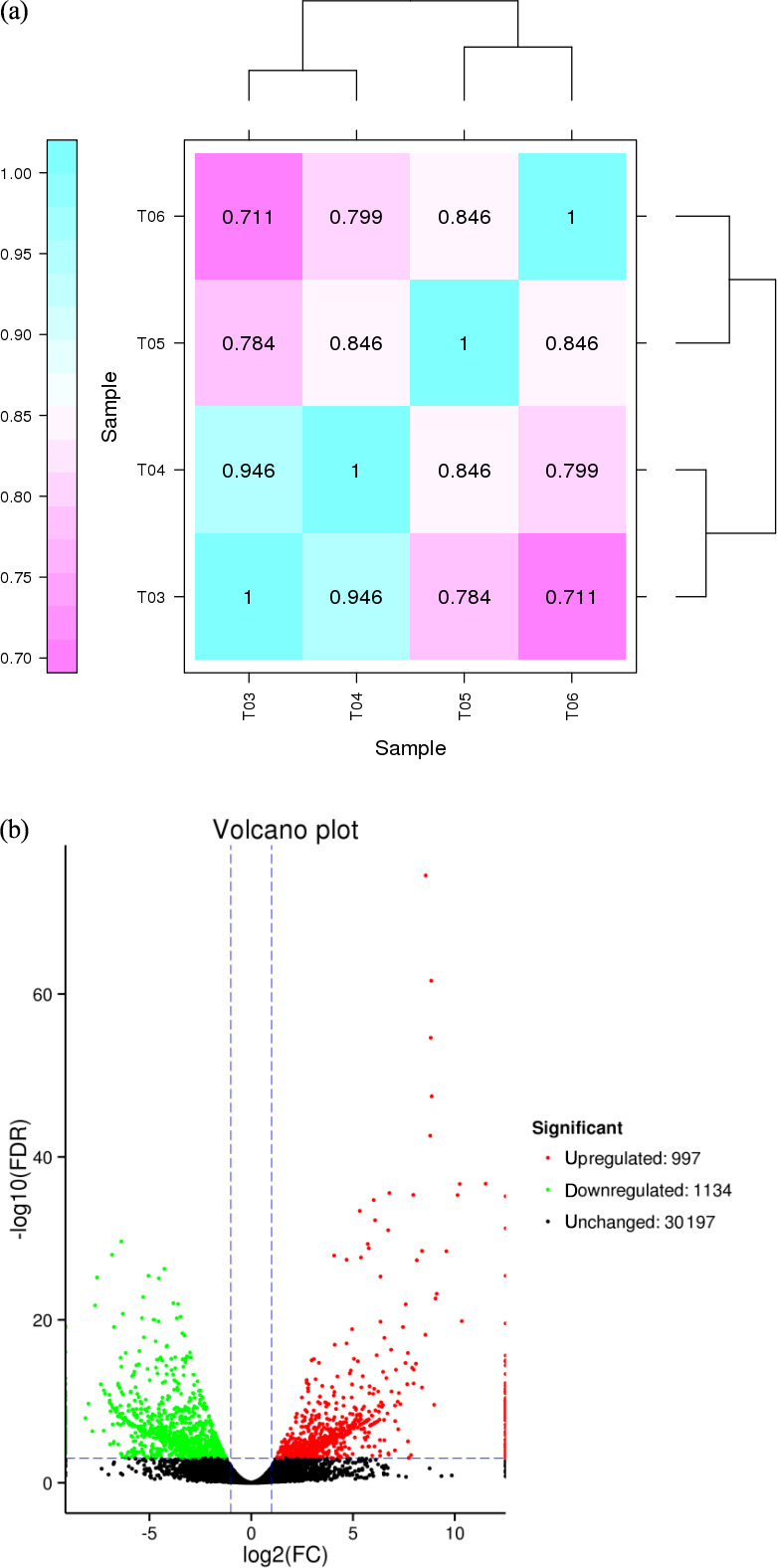
**(a)** Correlation heat map of gene expression level in four samples. Notes: T03 and T04 represent two biological replicates of Pekin ducks; T05 and T06 represent two biological replicates of mulard ducks. **(b)** Volcano plot of differentially expressed genes The abscissa is the value of log2 (FPKM), ie, the logarithm of the mean value of expression in both samples; the ordinate is the value of log2 (FC), the logarithm of the difference in gene expression between two samples which used to measure differences in expression. Each dot represents a gene. Green dot: genes with significantly downregulated expression; red dot: genes with significantly upregulated expression; black dot: genes without differential expression. The expression level of the upregulated gene in mulard ducks is higher than that in Pekin ducks; the opposite is the case for the downregulated gene.

FPKM (fragments per kilobase of transcript per million mapped reads) is a commonly used method to estimate gene expression levels in transcriptome data analyses, and the Pearson correlation coefficient, R, is used as an evaluation index of biological replicate correlation (Schulze et al., 2012). A correlation diagram of gene expression for each pair of biological replicate samples under the same condition was calculated (Fig. 2a), and the reliability and repeatability of the test were very high.

**Figure 3:**
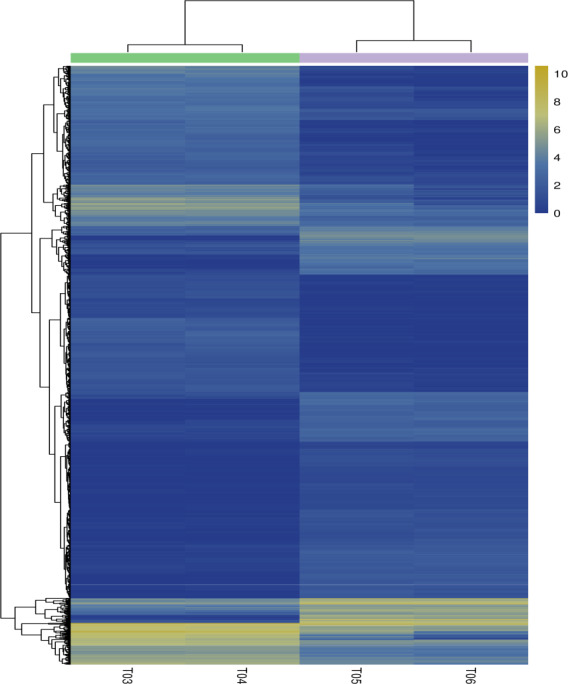
Heatmap of the differentially expressed genes. Columns indicate individual samples, and the row represents each differentially expressed genes. The color scale represents log10 (FPKM). Notes: T03 and T04 represent two biological replicates of Pekin ducks; T05 and T06 represent two biological replicates of mulard ducks.

The false discovery rate (FDR) was used as a key indicator of the integrity of the DEG dataset, with 2131 transcripts identified as differentially expressed between the two duck breeds with the standard FDR < 0.001 and FC > 2.0. The DEG dataset included 997 genes that were more highly expressed in the mulard duck than in the Pekin duck and 1134 that were more highly expressed in the Pekin duck than in the mulard duck (File S1 in the Supplement). The top 20 enriched DEGs, i.e., more highly expressed, in the mulard duck, included calmodulin, CALM and DS3 (Table 3), whereas the top 20 enriched DEGs in the Pekin duck included CAMK4, CPCX1 and coiled-coil domain-containing protein 70 (Table 4). The genes more highly expressed in the mulard rather than in the Pekin duck were the regulator of G-protein signaling 2, a voltage-dependent calcium channel L type alpha-1D (CACNA1D), the ryanodine receptor 2 (RYR2), calmodulin (CALM), argininosuccinate synthase, delta-1-pyrroline-5-carboxylate synthetase ALDH18A1 (P5CS) and proline dehydrogenase (PRODH). Genes more highly expressed in the Pekin duck as compared to those of the mulard included zona pellucida sperm-binding protein 4, testis-specific serine/threonine-protein kinase 3, tyrosine 3-monooxygenase, mast/stem cell growth factor receptor Kit, Kelch-like protein 10 isoform X1, 1-phosphatidylinositol 4,5-bisphosphate phosphodiesterase zeta-1, aryl hydrocarbon receptor, voltage-dependent calcium channel T type alpha-1I (CACNA1I), phosphatidylinositol phospholipase C zeta (PLCZ), phospholamban (PLN), phosphorylase kinase alpha/beta subunit (PHK), calcium/calmodulin-dependent protein kinase IV (CAMK4), calcium/calmodulin-dependent 3${}^{\prime }$,5${}^{\prime }$-cyclic nucleotide phosphodiesterase (PDE1), creatine kinase, long-chain-fatty-acid–CoA ligase (ACSBG), aquaporin-7 (AQP7) and glycerol kinase GlpK (GK). These differences were also notable in terms of global analyses, in which the distributions of gene expression differences between the mulard and Pekin duck testis samples were distinct (Fig. 2b). Clustering analyses further demonstrated good reproducibility and reasonable grouping of the DEG dataset (Fig. 3).

**Table 3:** The top 20 DEGs found to be enriched, i.e., more highly expressed, in the mulard duck.

Gene_ID	T03_FPKM	T04_FPKM	T05_FPKM	T06_FPKM	FDR	log2FC	Regulated
c259764.graph_c0	0.300252	0	175.8767	248.8426	1.42 × 10${}^{-20}$	10.34449418	Up
c102826.graph_c0	0	0.044617	29.42121	26.23158	2.11 × 10${}^{-37}$	10.24640925	Up
c217539.graph_c0	0	0.041665	11.25527	12.3068	6.25 × 10${}^{-24}$	9.111506603	Up
c230170.graph_c0	0.12502	0.08892	51.98331	52.81251	2.33 × 10${}^{-62}$	8.844165068	Up
c260971.graph_c1	0.680406	0.120984	225.9272	168.5905	2.38 × 10${}^{-55}$	8.816421563	Up
c264446.graph_c0	0	0.115006	24.53546	19.992	6.75 × 10${}^{-19}$	8.555827408	Up
c254097.graph_c0	0.165819	0.196564	65.71607	42.92932	4.80 × 10${}^{-28}$	8.134711944	Up
c257674.graph_c0	0	0.077763	9.803205	10.33076	1.30 × 10${}^{-14}$	7.983271223	Up
c263836.graph_c0	0	0.035586	2.711423	5.582583	0.000396	7.851213574	Up
c103252.graph_c0	0	0.061355	7.649744	5.202753	7.83 × 10${}^{-13}$	7.664124651	Up
c245722.graph_c0	0	0.058163	5.237366	6.192476	1.23 × 10${}^{-22}$	7.588975803	Up
c250240.graph_c0	0.018876	0.013426	3.124597	2.922019	7.58 × 10${}^{-20}$	7.45381347	Up
c276764.graph_c1	6.905169	7.498273	1517.476	838.3763	9.69 × 10${}^{-12}$	7.253456689	Up
c271910.graph_c2	0.645458	0.879901	141.716	76.39715	3.33 × 10${}^{-10}$	7.064349354	Up
c262074.graph_c0	0.126387	0	9.71331	6.860314	6.26 × 10${}^{-12}$	6.891966036	Up
c247209.graph_c0	0.129116	0	7.887562	8.630786	4.67 × 10${}^{-17}$	6.869811596	Up
c265640.graph_c0	0.303276	0.323555	43.02991	30.739	2.73 × 10${}^{-36}$	6.785737723	Up
c257878.graph_c0	0	0.056662	2.197879	3.923914	0.000237	6.738106642	Up
c240461.graph_c0	0.255645	0.212131	23.88739	28.60951	9.89 × 10${}^{-32}$	6.727028981	Up
c243292.graph_c0	0.158901	0	9.133037	7.187676	1.62 × 10${}^{-18}$	6.542796185	Up

**Table 4:** The top 20 DEGs found to be enriched, i.e., more highly expressed, in the Pekin duck.

Gene_ID	T03_FPKM	T04_FPKM	T05_FPKM	T06_FPKM	FDR	log2FC	Regulated
c197407.graph_c0	4.150825	5.81225	0.255614	0	2.19 × 10${}^{-8}$	-5.423989078	Down
c255508.graph_c0	272.7484	206.052	7.495369	3.982625	6.79 × 10${}^{-17}$	-5.49261772	Down
c216167.graph_c0	3.503095	3.683175	0	0.153099	1.45 × 10${}^{-7}$	-5.57510716	Down
c213220.graph_c0	42.23808	25.56573	1.576416	0	2.17 × 10${}^{-6}$	-5.586177912	Down
c266403.graph_c0	4.286627	9.875276	0.206031	0.084076	0.000318	-5.700472831	Down
c234781.graph_c0	3.565642	2.983583	0.137774	0	5.62 × 10${}^{-9}$	-5.72286459	Down
c205824.graph_c0	60.33108	29.98383	1.661492	0.188336	0.000528	-5.760264271	Down
c186936.graph_c0	11.88476	7.719809	0.358473	0	5.51 × 10${}^{-8}$	-5.931096357	Down
c234673.graph_c1	7.375337	16.70711	0.348416	0.050778	0.000162	-6.026948944	Down
c269805.graph_c1	1.764625	1.458607	0.046992	0	1.71 × 10${}^{-8}$	-6.252171838	Down
c249220.graph_c0	2.273626	3.0725	0.074674	0	1.39 × 10${}^{-11}$	-6.302035053	Down
c186446.graph_c0	11.48321	15.84912	0.244637	0.124787	1.79 × 10${}^{-21}$	-6.305891784	Down
c245393.graph_c0	15.39968	18.77265	0.370987	0.075695	2.43 × 10${}^{-30}$	-6.378435949	Down
c264196.graph_c1	48.64439	35.18299	0.736622	0.325645	4.56 × 10${}^{-16}$	-6.418455493	Down
c213765.graph_c0	7.572708	13.37683	0.244637	0	6.88 × 10${}^{-9}$	-6.5541524	Down
c277048.graph_c0	11.80391	10.53549	0	0.23265	6.41 × 10${}^{-10}$	-6.611671805	Down
c210720.graph_c0	3.225231	2.574819	0.064854	0	1.86 × 10${}^{-9}$	-6.635780171	Down
c276524.graph_c0	238.9048	176.4517	2.980127	1.234535	7.56 × 10${}^{-20}$	-6.740265327	Down
c211503.graph_c0	35.61218	42.42217	0.753441	0	1.02 × 10${}^{-28}$	-6.837800434	Down
c247833.graph_c0	2.17709	4.000148	0.04469	0	4.04 × 10${}^{-7}$	-7.244011346	Down

### 3.4 GO function enrichment

**Figure 4:**
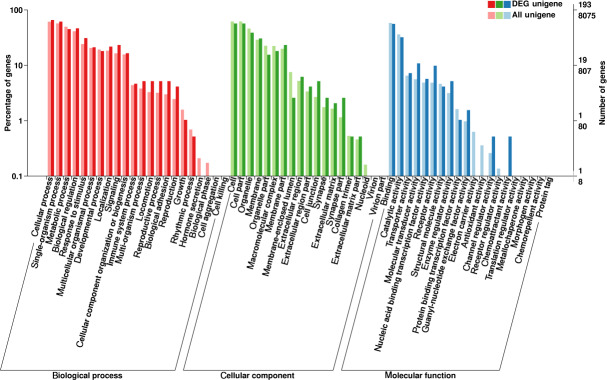
GO annotation of differentially expressed genes. The abscissa is the GO classification, the left vertical axis is the percentage of the number of genes and the right is the number of genes.

The present study used GO database analyses to compare and annotate DEGs into the three major classifications of biological process, molecular function and cellular component, resulting in the functional annotation of 786 DEGs (Fig. 4). The three major classifications were further divided into a total of 61 specific categories, consisting of 22, 19 and 20 categories, respectively (Fig. 4). The number of DEGs was most abundant in the cellular and single-organism processes within the biological process classification, in cell and cell-part categories of the cellular component classification, and in binding and catalytic activity in the molecular function classification (Fig. 4). Of note, within the biological process classification, abundant DEGs were found within categories related to reproduction and reproductive processes.

### 3.5 KEGG pathway enrichment analysis

**Figure 5:**
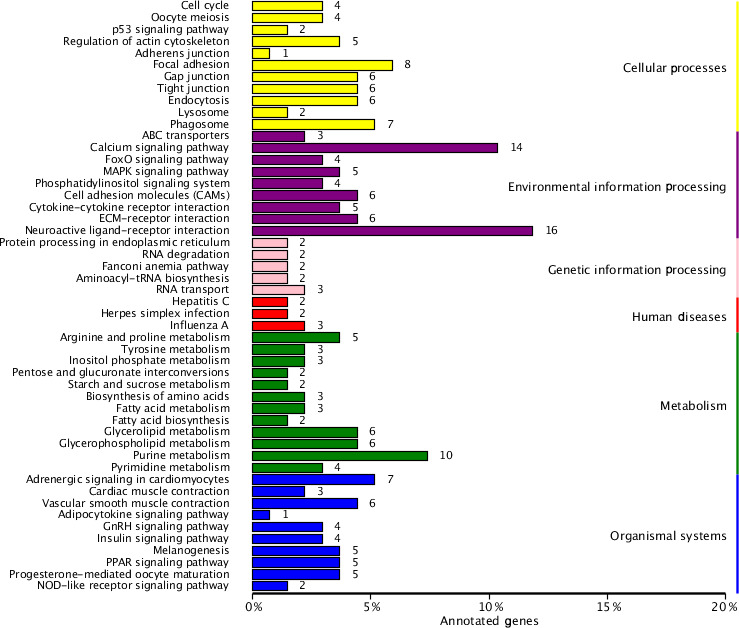
List of KEGG pathways for differentially expressed genes. The ordinate is the name of the KEGG metabolic pathway, and the abscissa is the ratio of the number of genes annotated to pathways and the ratio of the number to the total number of genes annotated.

To identify the main biochemical and signaling pathways in the DEG dataset, KEGG analyses were performed, resulting in the classification of DEGs into 93 signaling pathways. The 10 pathways were significant at P<0.05, and the most significant enrichment occurred in pathways related to neuroactive ligand–receptor interactions, followed by calcium signaling pathways, purine metabolism and glycerolipid metabolism signaling pathways. Classification of the DEG dataset according to gene annotation and KEGG pathways (Fig. 5; Table 5) indicated significant enrichment in pathways related to the reproductive processes included in calcium signaling and the peroxisome proliferator-activated receptor (PPAR; nuclear hormone receptors activated by fatty acids) signaling pathways (Fig. 5). Six downregulated genes, including CACNA1I, PLCZ, PLN, PHKA_B, CAMK4 and PDE1, and three upregulated genes, CACNA1D, RYR2 and CALM, which participate in the calcium signaling pathway, were screened. Three downregulated genes, including E2.7.3.2, ACSBG and AQP7, and the upregulated gene for argininosuccinate synthase were screened in the PPAR signaling pathway. Additionally, the DEG dataset showed different degrees of enrichment particularly for those associated with signaling pathways involved in reproduction. Examples include portions of the mitogen-activated protein kinase (MAPK) signaling pathways, the gonadotropin-releasing hormone (GnRH) signaling pathway and the Wnt signaling pathways, which are used in cell–cell communication and same-cell communication and which are implicated in a range of developmental processes, including cell fate, patterning and embryonic development.

**Table 5:** List of KEGG pathway for differentially expressed genes.

Pathway	UniGene	Related UniGene	P value
	number	number	
Neuroactive ligand–receptor interaction	16	297	0.000358
Calcium signaling pathway	14	256	0.000732
Purine metabolism	10	216	0.013191
Glycerolipid metabolism	6	103	0.018991
Hepatitis C	2	11	0.020433
Glycerophospholipid metabolism	6	110	0.025347
Ovarian steroidogenesis	1	2	0.040695
Gap junction	6	124	0.041997
PPAR signaling pathway	5	95	0.045417
Arginine and proline metabolism	5	97	0.048928
Fatty acid biosynthesis	2	18	0.051776
Vascular smooth muscle contraction	6	141	0.069851
Progesterone-mediated oocyte maturation	5	110	0.075565
ABC transporters	3	55	0.10318
Adrenergic signaling in cardiomyocytes	7	194	0.104117
Phagosome	7	196	0.108383
Melanogenesis	5	126	0.117174
Leukocyte transendothelial migration	1	6	0.117218
Tyrosine metabolism	3	59	0.120712
Cell adhesion molecules (CAMs)	6	165	0.123714

### 3.6 Verified by qRT-PCR

**Figure 6:**
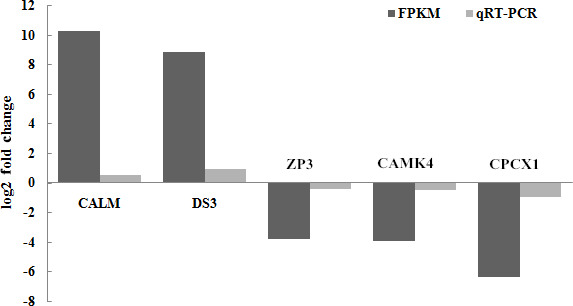
Validation of the RNA-seq data by real-time PCR for selected genes. FPKM is a commonly used method to estimate gene expression levels in transcriptome data analysis; log2 (fold change) is the logarithm of the difference in gene expression between two samples which is used to measure differences in expression.

A set of five genes, corresponding to β-actin (CALM, DS3, ZP3, CAMK4 and CPCX1), were selected for the verification of the differential expression by qRT-PCR, as detailed in Sect. 2. Of these genes, CALM and DS3 were more highly expressed in the mulard duck compared to that of the Pekin duck, whereas the remaining three candidates were more highly expressed in the Pekin duck, as observed in the transcriptome results described above. CAMK4, CALM and ZP3 genes are the key significant differential genes related to reproduction and development, which will serve as the basis for further research on the differential genes; with FDR (false discovery rate) as the key indicator for screening differentially expressed genes, DS3 and CPCX1 are the most significantly enriched in mulard duck and Pekin duck to effectively verify the results of transcriptome sequencing. The qRT-PCR analyses of these genes using independent biological samples confirmed the transcriptomic results, with the expression levels of CALM and DS3 in mulard ducks being higher than those in Pekin ducks, whereas the expression levels of ZP3, CAMK4 and CPCX1 were higher in Pekin ducks than in mulard ducks (Fig. 6).

## 4 Discussion

The present study utilized transcriptome sequencing to yield a set of DEGs comparing the testes of mulard and Pekin ducks to elucidate gene regulatory and expression pathways that might be linked to the sterile phenotype, that is to say abnormal testicular development in this study. Our analyses yielded 2131 DEGs that were analyzed by KEGG classification with significant enrichment seen in a range of biochemical metabolic and signaling pathways and with some DEGs identified as being closely associated with the reproductive process and development.

Although the development and differentiation processes leading to the mature and functional male reproductive system are complex, and our understanding is incomplete, some potentially interesting insights can be gained from our analyses. At present, various studies have shown that both p53 (Li et al., 2013) and Wnt metabolic signaling pathways (Zheng et al., 2014) contribute to male reproductive development. The MAPK extracellular signal-regulated protein kinase (MEK)/serine-threonine protein kinase (MOS)/MAPK signaling pathway has been suggested as being involved in mammalian spermatogenesis, proper meiotic (re)initiation, and subsequent aspects of mitosis and mediating cross talk to the maturation promoting factor (Almog et al., 2008; Show et al., 2008; Sun et al., 1999). Other participants identified in the mulard DEG dataset included the GnRH and its downstream signaling pathways that regulate the secretion of reproductive hormones through the gonadal axis in animals coupled with hypothalamic neurohormones that promote the release of gonadotropins from the pituitary to regulate endocrinological and developmental aspects of animal reproduction (Lee et al., 2008).

Santi et al. (1998) found that calcium (${\mathrm{Ca}}^{2+}$) is an important secondary messenger in eukaryotic cells, and calcium signaling and calcium flux participate in diverse cellular processes, including spermatogenesis and the physiological activity (motility and response) of sperm. Calcium signaling also regulates phospholipase 2 (PLA2), which in turn plays an important role during the acrosome reaction that is critical for the male reproductive processes of sperm capacitation and sperm–egg fusion. The activation and regulation of sperm PLA2 activity are also mediated by specific G-protein-coupled receptors according to Yuan et al. (2003). Within this context, we identified six related genes (CACNA1I, PLCZ, PLN, PHKA/B, CAMK4 and PDE1) that were significantly more abundant in the Pekin duck than in the mulard duck, and three genes (CACNA1D, RYR2 and CALM) were more abundant in the mulard duck testis tissues than in those of the Pekin duck. Phospholipase C zeta (PLCZ) plays an important role during spermatogenesis, sperm–egg fusion, embryonic development and the catalyzation of reactions (Giesecke et al., 2010; Swann et al., 2006). Le Naour et al. (2000) showed that PLCZ activity not only affects fertilization but also plays an important role in embryonic development, particularly in promoting egg division during early embryonic development into the blastocyst stage. The loss of PLCZ expression in mulard duck testes may be a critical factor contributing to the defects related to the response to spermatogenesis, normal embryonic development, sperm–egg combination and other reproductive processes which lead to infertility in these ducks. The calcium-calmodulin-dependent protein kinase IV (CAMK4) is known to play an important role in cell fate and germ cell development. CAMK4 contributes to the regulation of meiosis, chromosome pairing and the maintenance of genome integrity during germ cell genesis and development. Therefore, the decreased relative expression of CMK4 seen in the mulard duck may result in impaired meiosis and germ cell formation. One potential mechanism by which reduced CAMK4 may act is via its regulation of protamine by phosphorylation, a reaction required for normal spermatozoal functioning (Dada et al., 2012). PPAR has pleiotropic functions including that of the process of mammalian reproduction and the regulation of spermatogenesis (Kobayashi et al., 2003). Loss of PPAR activity may lead to dysfunctions in the interstitial Leydig cells that produce testosterone in response to the luteinizing hormone leading to developmental defects in the androgen-dependent male reproductive system, including normal testicular function in adults, which negatively affects spermatogenesis and fertility. Generally, it is believed that steroid production is regulated by the trophic hormone, which promotes the transport of cholesterol from storage and synthesis sites to the mitochondrial inner membrane. Zhao et al. (2005) showed that peroxisome proliferators can block the transport induced by trophic hormones and thereby affect the male reproductive system. Our data showed that the DEGs corresponding to the PPAR pathway in the testes of mulard ducks were significantly enriched (in this instance expressed at a much lower level) as compared to those of Pekin ducks. This suggests that the lack of or decreased function of this pathway may contribute to the reproductive disorders seen in mulard ducks. This study screened and selected three downregulated genes (GK; long-chain-fatty-acid–CoA ligase, ACSBG; aquaporin-7, AQP7) and one upregulated gene (argininosuccinate synthase, AS). Among the DEGs screened in this pathway. Suzuki-Toyota et al. (1999) suggested that AQP7 is significantly expressed on the plasma membrane of sperm in the testis and epididymis. The expression of AQP7 in spermatozoa is known to affect semen quality. Low expression of the AQP7 gene in the testes of mulard ducks may greatly affect sperm production or quality, resulting in abnormal development of the testes in mulard ducks.

Some pathways that appeared abnormally expressed in the mulard duck were found that did not appear to have direct links to fertility. These included various neuroactive ligand–receptor interactions. We speculate that these may be involved in nerve body development, which may have indirect effects in terms of behavior and the activities and function of the testes. Additional pathways identified included that of purine/glycerolipid metabolism, which may function to provide nucleotide pools and signaling events critical to testis function.

## 5 Conclusion

A set of DEGs was identified in a comparative analysis of gene expression between testis tissues of mulard and Pekin ducks. Differential regulation of genetic pathways involved in spermatogenesis, meiosis and sperm–egg fusion was observed in mulard ducks, which provides candidate networks and hypotheses to account for the male-sterile phenotype of mulard ducks. Our data provided a foundation for further functional studies to elucidate the mechanisms of hybrid sterility, probe normal testicular development and function, and provide a means for therapeutic targeting to overcome the lack of reproductive vigor observed in the mulard duck.

## Data Availability

The original data have been uploaded to the Sequence Read Archive (SRA) database (, accession: PRJNA657980, Fujian Academy of Agricultural Sciences, 2020).
